# Identification of COVID-19 patients at risk of hospital admission and mortality: a European multicentre retrospective analysis of mid-regional pro-adrenomedullin

**DOI:** 10.1186/s12931-022-02151-1

**Published:** 2022-08-28

**Authors:** Emanuela Sozio, Nathan A. Moore, Martina Fabris, Andrea Ripoli, Francesca Rumbolo, Marilena Minieri, Riccardo Boverio, María Dolores Rodríguez Mulero, Sara Lainez-Martinez, Mónica Martínez Martínez, Dolores Calvo, Claudia Gregoriano, Rebecca Williams, Luca Brazzi, Alessandro Terrinoni, Tiziana Callegari, Marta Hernández Olivo, Patricia Esteban-Torrella, Ismael Calcerrada, Luca Bernasconi, Stephen P. Kidd, Francesco Sbrana, Iria Miguens, Kirsty Gordon, Daniela Visentini, Jacopo M. Legramante, Flavio Bassi, Nicholas Cortes, Giorgia Montrucchio, Vito N. Di Lecce, Ernesto C. Lauritano, Luis García de Guadiana-Romualdo, Juan González del Castillo, Enrique Bernal-Morell, David Andaluz-Ojeda, Philipp Schuetz, Francesco Curcio, Carlo Tascini, Kordo Saeed

**Affiliations:** 1 Infectious Disease Unit, Azienda Sanitaria Universitaria Integrata di Udine (ASU FC), 33100 Udine, Italy; 2 Department of Microbiology, Basingstoke and North Hampshire Hospital, Hampshire Hospitals NHS Foundation Trust, Basingstoke, UK; 3 Institute of Clinical Pathology, Azienda Sanitaria Universitaria Integrata di Udine (ASU FC), 33100 Udine, Italy; 4 Fondazione Toscana “Gabriele Monasterio”, 56124 Pisa, Italy; 5 Clinical Biochemistry Laboratory, Città Della Salute e della Scienza Hospital, University of Turin, 10126 Turin, Italy; 6 Department of Experimental Medicine, University of Rome Tor Vergata, Rome, Italy; 7 Unit of Laboratory Medicine, Tor Vergata University Hospital, Rome, Italy; 8 Emergency Medicine, Azienda Ospedaliera Nazionale Santi Antonio e Biagio e Cesare Arrigo, Alessandria, Italy; 9 Critical Care Unit, Hospital Universitario Santa Lucía, Cartagena, Spain; 10 Emergency Department, Hospital Clínico San Carlos, Instituto de Investigación Sanitaria San Carlos, Madrid, Spain; 11 Infectious Disease Unit, Hospital Universitario Reina Sofía, Murcia, Spain; 12 Laboratory Medicine Department, Hospital Clínico Universitario, Valladolid, Spain; 13 Medical University Department of Internal Medicine, Cantonal Hospital Aarau, Aarau, Switzerland; 14 Department of Surgical Sciences, University of Turin, Turin, Italy; 15 Department of Anesthesia, Intensive Care and Emergency, ‘Città della Salute e della Scienza’ Hospital, Turin, Italy; 16 Clinical Pathology Laboratory, Azienda Ospedaliera Nazionale Santi Antonio e Biagio e Cesare Arrigo, Alessandria, Italy; 17 Pneumology Department, Hospital Universitario Santa Lucía, Cartagena, Spain; 18 Laboratory Medicine Department, Hospital Universitario Reina Sofía, Murcia, Spain; 19 Primary Care Medina del Campo Urbano Area, Medina del Campo, Valladolid, Spain; 20 Institute of Laboratory Medicine, Cantonal Hospital Aarau, Aarau, Switzerland; 21 Emergency Service, University Hospital Gregorio Marañón and “Gregorio Marañón” Health Research Institute (IISGM), Madrid, Spain; 22 Department of Biochemistry, Basingstoke and North Hampshire Hospital, Hampshire Hospitals NHS Foundation Trust, Basingstoke, UK; 23 Department of Systems Medicine, University of Rome Tor Vergata, Rome, Italy; 24 Emergency Department, Tor Vergata University Hospital, Rome, Italy; 25 Department of Anesthesia and Intensive Care Medicine, Azienda Sanitaria Universitaria Integrata di Udine (ASU FC), 33100 Udine, Italy; 26 Faculty of Medicine, University of Southampton, Southampton, UK; 27 Laboratory Medicine Department, Hospital Universitario Santa Lucía, Cartagena, Spain; 28 Instituto Murciano de Investigación Biosanitaria (IMIB), Murcia, Spain; 29 Department of Intensive Care, Hospital Universitario HM Sanchinarro, Madrid, Spain; 30 Medical Faculty, University of Basel, Basel, Switzerland; 31 Department of Medicine (DAME), University of Udine, 33100 Udine, Italy; 32 Department of Infection, Southampton General Hospital, University Hospital Southampton NHS Foundation Trust, Tremona Road, Southampton, UK

**Keywords:** MR-proADM, SARS-CoV-2, Mortality, Hospital admission, Emergency department

## Abstract

**Background:**

Mid-Regional pro-Adrenomedullin (MR-proADM) is an inflammatory biomarker that improves the prognostic assessment of patients with sepsis, septic shock and organ failure. Previous studies of MR-proADM have primarily focussed on bacterial infections. A limited number of small and monocentric studies have examined MR-proADM as a prognostic factor in patients infected with SARS-CoV-2, however there is need for multicenter validation. An evaluation of its utility in predicting need for hospitalisation in viral infections was also performed.

**Methods:**

An observational retrospective analysis of 1861 patients, with SARS-CoV-2 confirmed by RT-qPCR, from 10 hospitals across Europe was performed. Biomarkers, taken upon presentation to Emergency Departments (ED), clinical scores, patient demographics and outcomes were collected. Multiclass random forest classifier models were generated as well as calculation of area under the curve analysis. The primary endpoint was hospital admission with and without death.

**Results:**

Patients suitable for safe discharge from Emergency Departments could be identified through an MR-proADM value of ≤ 1.02 nmol/L in combination with a CRP (C-Reactive Protein) of ≤ 20.2 mg/L and age ≤ 64, or in combination with a SOFA (Sequential Organ Failure Assessment) score < 2 if MR-proADM was ≤ 0.83 nmol/L regardless of age. Those at an increased risk of mortality could be identified upon presentation to secondary care with an MR-proADM value of > 0.85 nmol/L, in combination with a SOFA score ≥ 2 and LDH > 720 U/L, or in combination with a CRP > 29.26 mg/L and age ≤ 64, when MR-proADM was > 1.02 nmol/L.

**Conclusions:**

This international study suggests that for patients presenting to the ED with confirmed SARS-CoV-2 infection, MR-proADM in combination with age and CRP or with the patient’s SOFA score could identify patients at low risk where outpatient treatment may be safe.

## Introduction

All infections have the potential to manifest into life-threatening conditions. Infections due to Severe Acute Respiratory Syndrome Coronavirus-2 (SARS-CoV-2) are not exempt from this. An early diagnosis and assessment of infection severity is therefore crucial in order to initiate triaging and appropriate therapeutic strategies. There have now been over 265 million cases worldwide of SARS-CoV-2 infection since the end of 2019. Whilst most cases are asymptomatic or defined by mild symptoms, up to 15% of all cases develop severe pathology [1, 2]. This large number of cases has resulted in substantial demand being placed upon healthcare systems and resulted in over 5.2 million deaths. In these circumstances, trying to determine those in whom admission can be safely avoided, those who need admission and those who need admission to higher level care facilities could become even more of a challenge to already stretched emergency clinical staff. The effect of this could be either unnecessary admission of patients with uncomplicated infections or inappropriate discharges. The use of biomarkers which have a high sensitivity for assessing disease severity and significantly increased during the initial stages of the disease development may therefore facilitate improved triaging and earlier therapeutic decisions.

The presence of SARS-CoV-2 within the endothelium can lead to a secondary endotheliitis that promotes an impairment of vascular blood flow, a pro-thrombotic state and vascular leakage [3]. The progressive multi-organ failure associated with SARS-CoV-2 mortality is driven in part by significant inflammation and microvascular thrombosis.

Recent studies, pre COVID-19, have shown mid-regional pro-adrenomedullin (MR-proADM) concentrations to be rapidly induced in the initial stages of sepsis development [4] and progression towards sepsis-related multiple organ failure [5, 6] and can assist triaging in the emergency department [7–10] and safely avoid admission. Adrenomedullin (ADM) is a potent vasodilatory peptide hormone produced by endothelial cells and plays a key role in reducing vascular permeability and promoting endothelial stability and integrity following severe infection [6]. Thus, ADM may also be of interest within COVID-19 induced endotheliitis. Recent small scale studies suggest that MR-proADM, a mostly inert fragment split from ADM may offer considerable value for predicting the risk of developing critical illness, disease progress and prognosis in patients with COVID-19 [11–19].

An observational retrospective multi-centre study with consistent outcome measures involving patients with COVID-19 presenting to the Emergency Departments of 10 hospitals in the United Kingdom, Italy, Spain and Switzerland predominantly during the first wave was therefore devised. This study aimed to assess the effectiveness of a number of biomarkers, both novel and established, and clinical scores, such as SOFA and National Early Warning Score 2 (NEWS2) scores, in COVID-19 patients in the acute setting to identify patients with uncomplicated infection wherein admission can safely be avoided and to identify those at increased risk of further disease progression and mortality.

## Methodology

### Study design and ethical approval

The 10 secondary or tertiary care centres involved were: Hampshire Hospitals NHS Foundation Trust, Azienda Sanitaria Universitaria Integrata di Udine, 'Città della Salute e della Scienza' Hospital, Turin, Policlinico di Tor Vergata di Roma, Ospedale Civile Santi Antonio e Biagio e Cesare Arrigo di Alessandria, Hospital Universitario Santa Lucía, Cartagena, Hospital Clínico San Carlos, IDISSC, Madrid, Hospital Universitario Reina Sofía, Murcia, Hospital Clínico Universitario de Valladolid, and Cantonal Hospital Aarau. This resulted in 1,861 patients eligible for inclusion.

Outcomes were assessed by the composite end points of no admission to hospital, admission to hospital with no mortality and admission with mortality at 28 days from diagnosis of COVID-19.

The individual probability of being discharged directly from ED or of being admitted to hospital, with or without risk of mortality due to COVID-19, was estimated with several different implementations of machine learning models based on multiclass random forest classifiers. Random forest algorithms were developed with 2 subgroups of patients. One group comprised 1,436 patients that included the 16 most frequently collected variables (Table 1). The second group consisted of 646 patients for whom it was possible to have additional data relating to clinical scores at presentation to the emergency department. The same model was applied to both subgroups in order to make the interpretation of the data more robust and to obtain additional information from those cases in which it was possible to evaluate the clinical scores at evaluation in ED.

**Table 1:** Analysis of variance on the three selected groups​​

	Not admitted (n = 158; 11.0%)	Admitted without event (n = 986; 68.7%)	Admitted with event (n = 292; 20.3%)	P
Age (years)	51.6 ± 12.8	62.5 ± 15.3*	71.3 ± 12^#^°	< 0.001
Male gender	82 (51.9%)	617 (62.6%)*	206 (70.6)^#^°	< 0.001
Creatinine (mg/dl)	0.80 [0.69–0.94]	0.96 [0.78–1.16]*	1.16 [0.87–1.62]^#^°	< 0.001
Platelets (/mmc)	233.99 ± 127.70	232.03 ± 96.86	210.23 ± 94.21^#^°	0.003
MR-proADM (nmol/L)	0.57 [0.48–0.71]	0.83 [0.63–1.16]*	1.33 [0.97–2.03]^#^°	< 0.001
WBC (/mmc)	5.40 [4.35–6.50]	6.44 [4.72–8.70]*	7.53 [5.28–10.88]^#^°	< 0.001
Lymphocytes (/mmc)	1.20 [0.80–1.61]	0.98 [0.70–1.33]*	0.57 [0.81–1.14]^#^°	< 0.001
LDH (U/L)	471 [392–599]	389 [276–555]*	510 [375–735]^#^°	< 0.001
PCT (mg/dl)	0.05 [0.03–0.08]	0.08 [0.04–0.14]*	0.18 [0.09–0.46]^#^°	< 0.001
CRP (mg/L)	19.65 [9.42–46.12]	60.07 [25–106.59]*	103.12 [55.67–176]^#^°	< 0.001
Cardiovascular disease	8 (5.1%)	216 (21.9%) *	102 (34.9%)^#^°	< 0.001
Chronic respiratory diseases	9 (5.7%)	148 (15.0%)*	65 (22.3%)^#^°	< 0.001
Diabetes	17 (10.8%)	175 (17.8%)	111 (38%)^#^°	< 0.001
Chronic kidney disease	2 (1.3%)	100 (10.1%)*	83 (28.4%)^#^°	< 0.001
Malignancy	6 (3.8%)	61 (6.2%)	28 (9.6%)	0.039
Hypertension	28 (17.7%)	455 (46.2%)*	184 (63%)^#^°	< 0.001

*: p < 0.05 post-hoc “not admitted” vs “admitted without event”; ^#^: p < 0.05 post-hoc “not admitted” vs “admitted with event”; °: p < 0.05 post-hoc “admitted without event” vs “admitted with event”

Ethical approval was sought from the relevant boards or governance bodies of each participating hospital. The manuscript was drafted according to the Standards for the Reporting of Diagnostic accuracy studies STARD criteria [20].

### Inclusion criteria

Symptomatic individuals presenting to hospital were eligible for inclusion following detection of SARS-CoV-2 by real‐time reverse-transcription PCR (RT-qPCR). Exclusion criteria included pregnancy and being younger than 18 years old.

### Data collection

Measurement of MR-proADM levels was performed on EDTA (Ethylenediaminetetraacetic acid) blood samples within 48 h of being taken on evaluation in ED (in line with manufacturer’s guidance stating a 72 h period of stability) using an immunoassay (B.R.A.H.M.S. KRYPTOR™, Thermo Fisher Scientific, Henningsdorf, Germany). Data collected included demographics, prior comorbidities, clinical outcomes such as admission and mortality at 28 days. Blood results including White Blood Cell Count (WBC), lymphocyte count, C-reactive Protein (CRP), Procalcitonin (PCT), lactate dehydrogenase (LDH), D-dimer measurements and the raw data to calculate clinical scores like NEWS2 and SOFA, were collected when these were performed at presentation to ED. All samples were analysed as per each site’s laboratory procedures.

### Statistical analysis

Variables were reported using mean ± standard deviation, median and interquartile range or proportion, depending on their distribution; accordingly, comparison between groups was performed with unpaired t-tests, Mann–Whitney U-tests or chi-square tests.

Analysis of Variance testing was performed on selected groups of patients, such as those not admitted, those who were admitted and did not die and those who were admitted and died. Where a significant difference between groups was found post-hoc pairwise analysis was performed with Bonferroni correction.

For initial analysis only variables with less than 20% missing data were included and a complete case analysis was used to construct a multiclass Random Forest classifier. However, to specifically assess the potential impact of clinical scoring systems that are used in common clinical practice these were also included in a subsequent complete-case analysis.

In order to predict the observed outcomes (no admission and admission with or without death) a multiclass random forest classifier was built. The variables to be included in the analysis were selected with the Boruta algorithm.[21] A ten-fold cross-validation procedure, repeated 50 times, was followed to choose the random forest hyperparameters and to assess predictive performance, on the basis of a ROC (receiver operating characteristic) curve analysis. An interpretation of the random forest algorithm was accomplished by computing a ranking of the predictor’s importance[22] and constructing conditional decision trees,[23] with the predicted classes as target variables. All analyses were performed with R.[24] A p-value < 0.05 was considered as statistically significant.

## Results

Once variables with missing data greater than 20% were omitted 1,436 symptomatic patients presenting to ED with a diagnosis of COVID-19 were selected, with patient demographics and biomarker levels being summarized in Table 1.

**Fig. 1:**
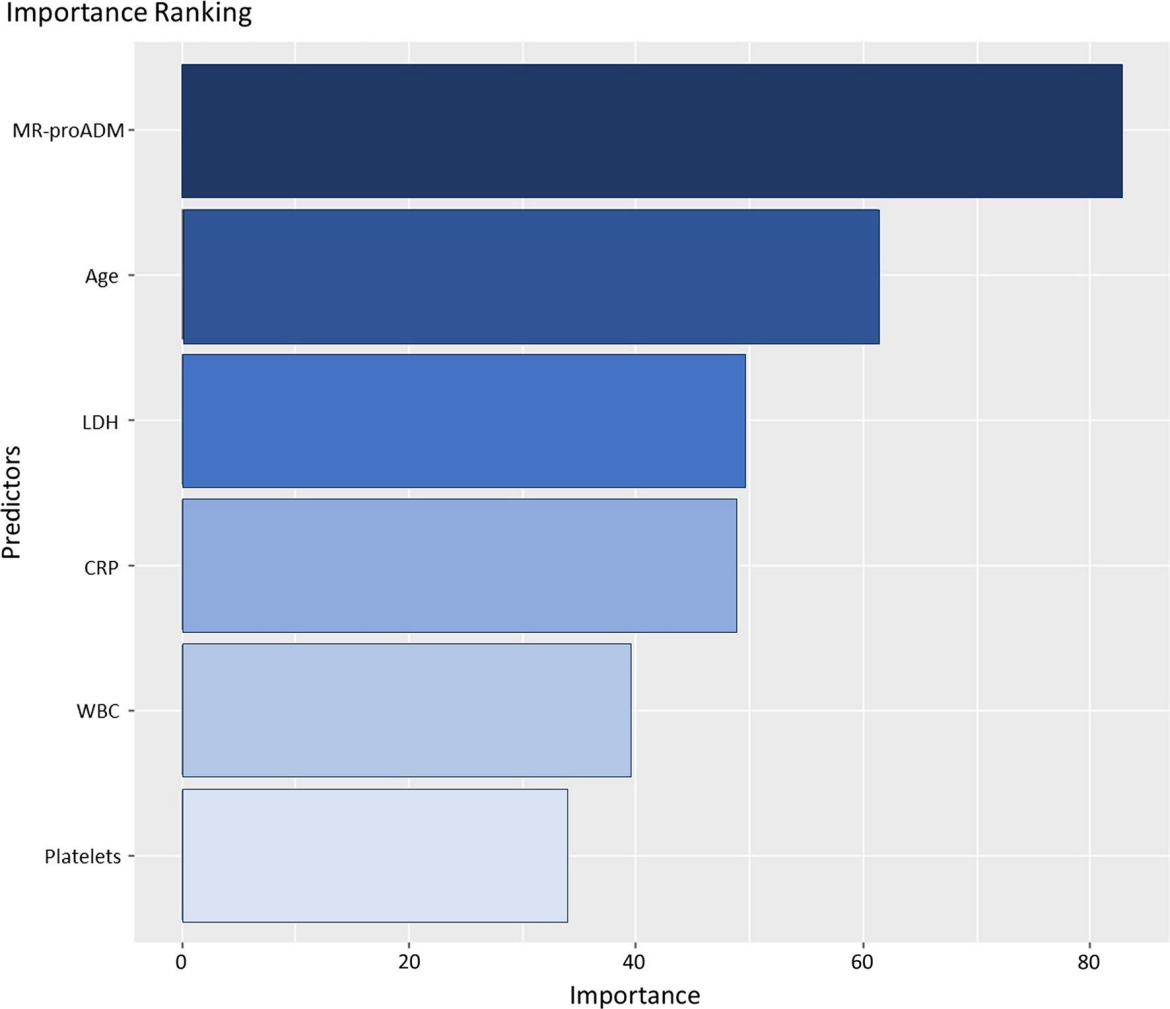
Importance ranking of predictors for the developed multiclass random forest classifier

To interpret the resultant random forest algorithm, predictors were then ranked and a decision tree built, as shown below. Multiclass random forest classifier furnished the ranking of importance for the predictor variables, as reported in Fig. 1: MR-proADM, LDH, CRP, age, WBC count and platelets were selected as variables, with MR-proADM being the most important variable as determined by the mean decrease in Gini index.

**Fig. 2:**
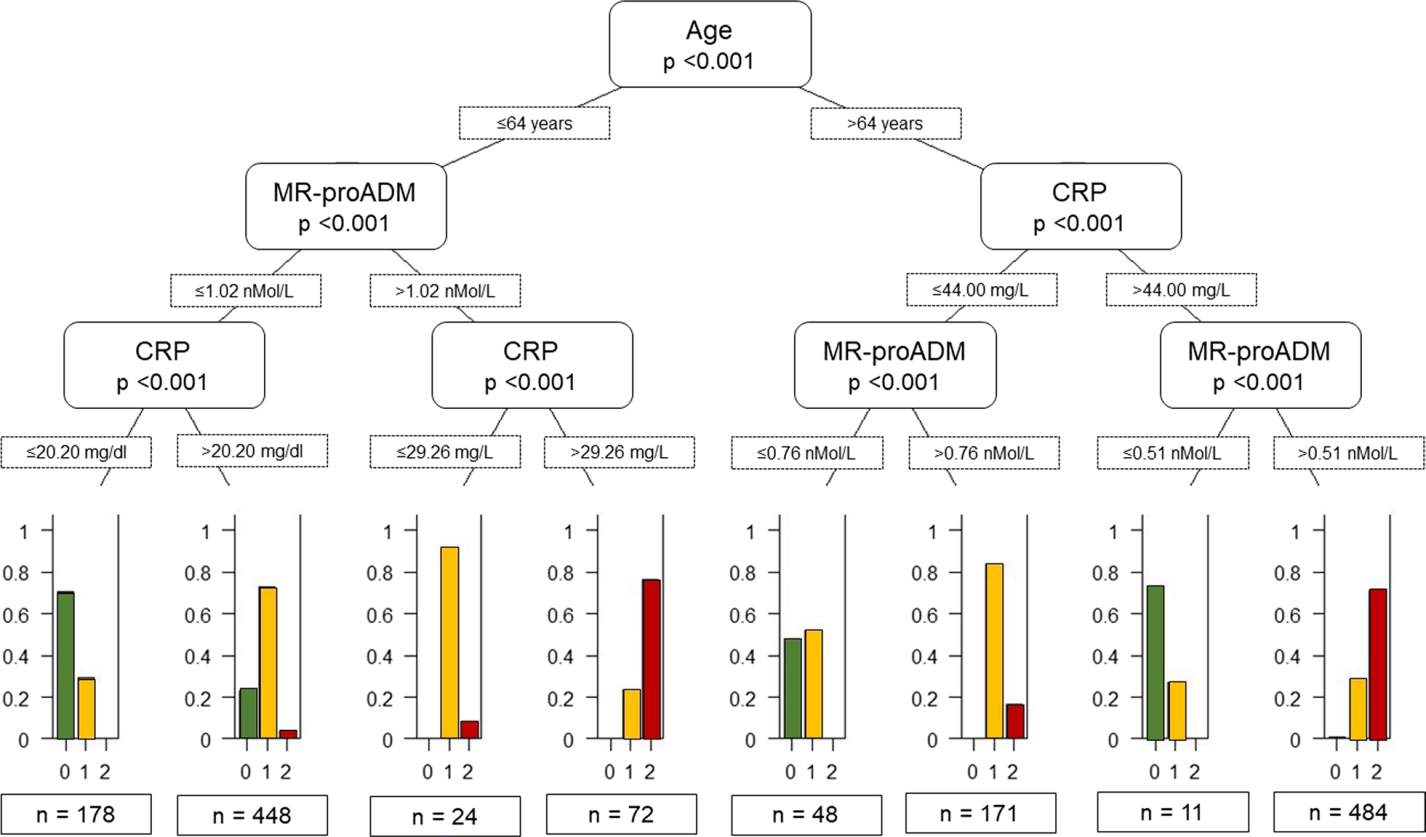
Conditional decision tree developed to explain the predictive performance of the multiclass random forest classifier

The decision tree in Fig. 2 allows an interpretation of the most important interactions captured by the random forest classifier. Age represents the predominant risk factor in determining the need for hospitalisation, which is further enhanced by MR-proADM and CRP measurements.

In patients ≤ 64 years old, if MR-proADM and CRP values were ≤ 1.02 nmol/L and < 20.20 mg/L, respectively, the risk of being admitted was minimal. On the other hand, for MR-proADM values > 1.02 nmol/L the risk of being hospitalised is high, which is compounded if a CRP value > 29.26 mg/L is added to this. Conversely, for those aged > 64 if CRP is ≤ 44 mg/L but pro-ADM > 0.76 nmol/L the probability of being hospitalised is high, whereas the probability of being hospitalised with risk of death is high when CRP is > 44 mg/L and MR-proADM is > 0.51 nmol/L.

**Fig. 3:**
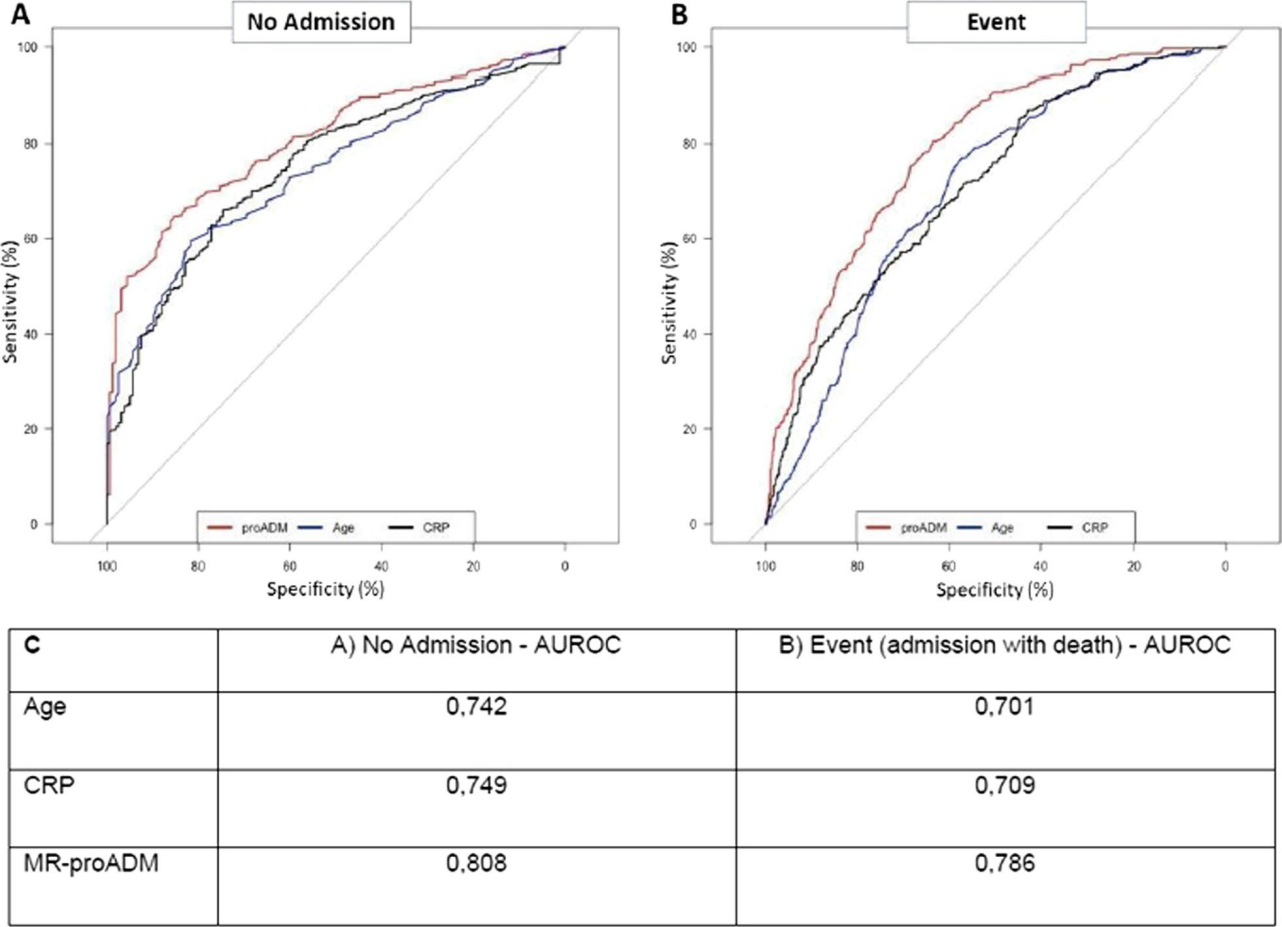
**A** ROC curve for admission avoidance, where clinical scores were not considered. **B** ROC curve for mortality, where clinical scores were not considered

The threshold values observed in the surrogate conditional decision tree shown in Fig. 2 are partially in agreement with a ROC analysis, shown below (Fig. 3), performed with classical statistical methods:

i. When considering age, for non-admitted patients the AUC was 0.742 and the best threshold was 61; for admitted patients who died the AUC was 0.701 and the best threshold was 64.
ii. Concerning CRP, the AUC for non-admitted patients was 0.749 and the best threshold was 45.13, whereas for admitted patients with poor outcome the AUC was 0.709 and the best threshold was 45.18.
iii. With regards to MR-proADM, the AUC for non-admitted patients was 0.808 and the threshold 0.771 and for patients admitted who died the AUC was 0.786 and the threshold was 0.911.

In order to evaluate whether the addition of clinical scores and D-dimer levels improved the predictive value to the model created 646 of the 1,861 initially eligible patients were selected, in whom this data was available. Patient demographics and biomarker values for this subgroup of 646 patients are summarized in Table 2.

**Table 2:** Analysis of variance on the three selected groups

	Not admitted (n = 131; 20.2%)	Admitted without event (n = 421; 65.2%)	Admitted with event (n = 94; 14.6%)	P
Age (years)	51.0 ± 12.3	65.6 ± 14.3*	75.1 ± 10.6^#^°	< 0.001
Male gender	67 (51.1%)	260 (61.8%)	56 (59.6%)	0.097
Creatinine (mg/dl)	0.79 [0.67–0.91]	0.95 [0.79–1.11]*	1.01 [0.8–1.46]^#^	< 0.001
Platelets (/mmc)	236.79 ± 136.01	244.93 ± 108.65	215.55 ± 104.54	0.106
MR-proADM (nmol/L)	0.57 [0.48–0.70]	0.91 [0.70 -1.26]*	1.345 [0.98–2.22]^#^°	< 0.001
WBC (/mmc)	5.30 [4.25–6.50]	6.24 [4.42–8.76] *	7.63 [5.20–11.04]^#^°	< 0.001
Lymphocytes (/mmc)	1.20 [0.80–1.70]	0.88 [0.62–1.20]*	0.77 [0.47–1.05]^#^°	< 0.001
LDH (U/L)	499 [418–621]	553 [418–694]*	735 [544–971]^#^°	< 0.001
Procalcitonin (mg/dl)	0.05 [0.03–0.08]	0.07 [0.04–0.14]*	0.13 [0.07–0.45]^#^°	< 0.001
CRP (mg/L)	20.10 [9.80–44.75]	59.45 [19.60–99.56]*	87.22 [48.27–149.70]^#^°	< 0.001
D-Dimer (ng/ml)	493 [350–676]	640 [428–1132]*	969 [516–1777]^#^°	< 0.001
Cardiovascular disease	4 (3.1%)	130 (30.9%)*	49 (52.1%)^#^°	< 0.001
Chronic respiratory disease	8 (6.1%)	71 (16.9%)*	28 (29.8%)^#^°	< 0.001
Diabetes	12 (9.2%)	28 (6.7%)	12 (12.8%)	0.125
Chronic kidney disease	0 (0.0%)	37 (8.8%) *	19 (20.2%)^#^°	< 0.001
Malignancy	4 (3.1%)	43 (10.2%)	13 (13.8%)^#^	0.012
Hypertension	23 (17.6%)	209 (49.6%) *	59 (62.8%)^#^	< 0.001
SOFA score	0 [0–1]	3 [2–4]*	4 [2–5]^#^°	< 0.001
NEWS2 score	0 [0–0]	1 [0–3]*	2 [0–4]^#^	< 0.001

*: p < 0.05 post-hoc “not admitted” vs “admitted without event”; ^#^: p < 0.05 post-hoc “not admitted” vs “admitted with event”; °: p < 0.05 post-hoc “admitted without event” vs “admitted with event”

With this subgroup the resultant random forest model had a sensitivity of 93.39 ± 1.53%, a specificity of 91.36 ± 1.45% and area under the curve of 95.9 ± 0.28% for those not requiring admission. For patients that died the random forest model had a sensitivity of 85.5 ± 2.86%, a specificity of 70.45 ± 3.79% and area under the curve of 79.37 ± 0.68%.

**Fig. 4:**
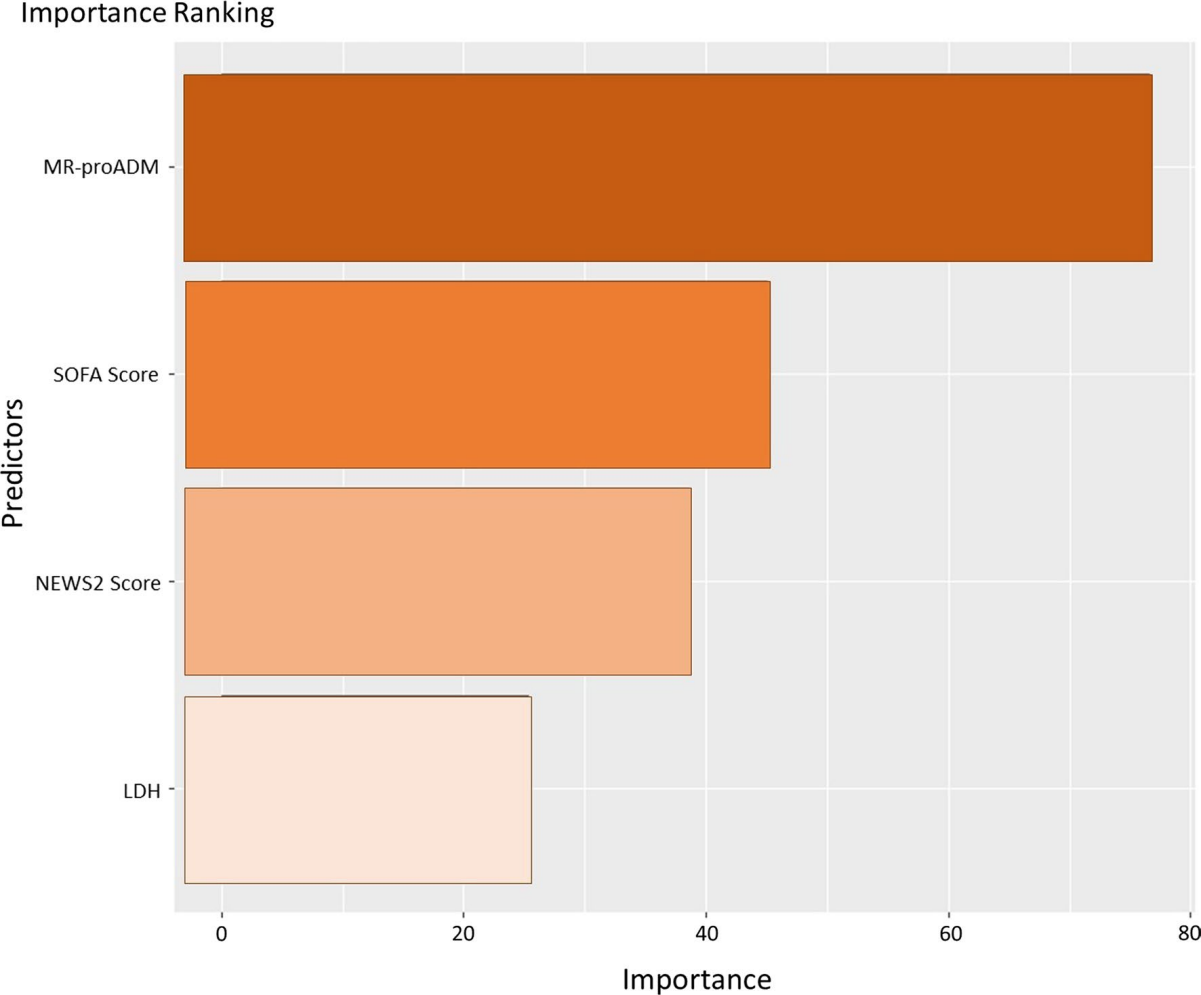
Importance ranking of predictors for the developed multiclass random forest classifier

In this case the multiclass random forest classifier furnished the ranking of importance for the predictor variables, as reported in Fig. 4: MR-proADM, LDH, SOFA and NEWS2 scores were selected, with MR-proADM still being the most important variable.

**Fig. 5:**
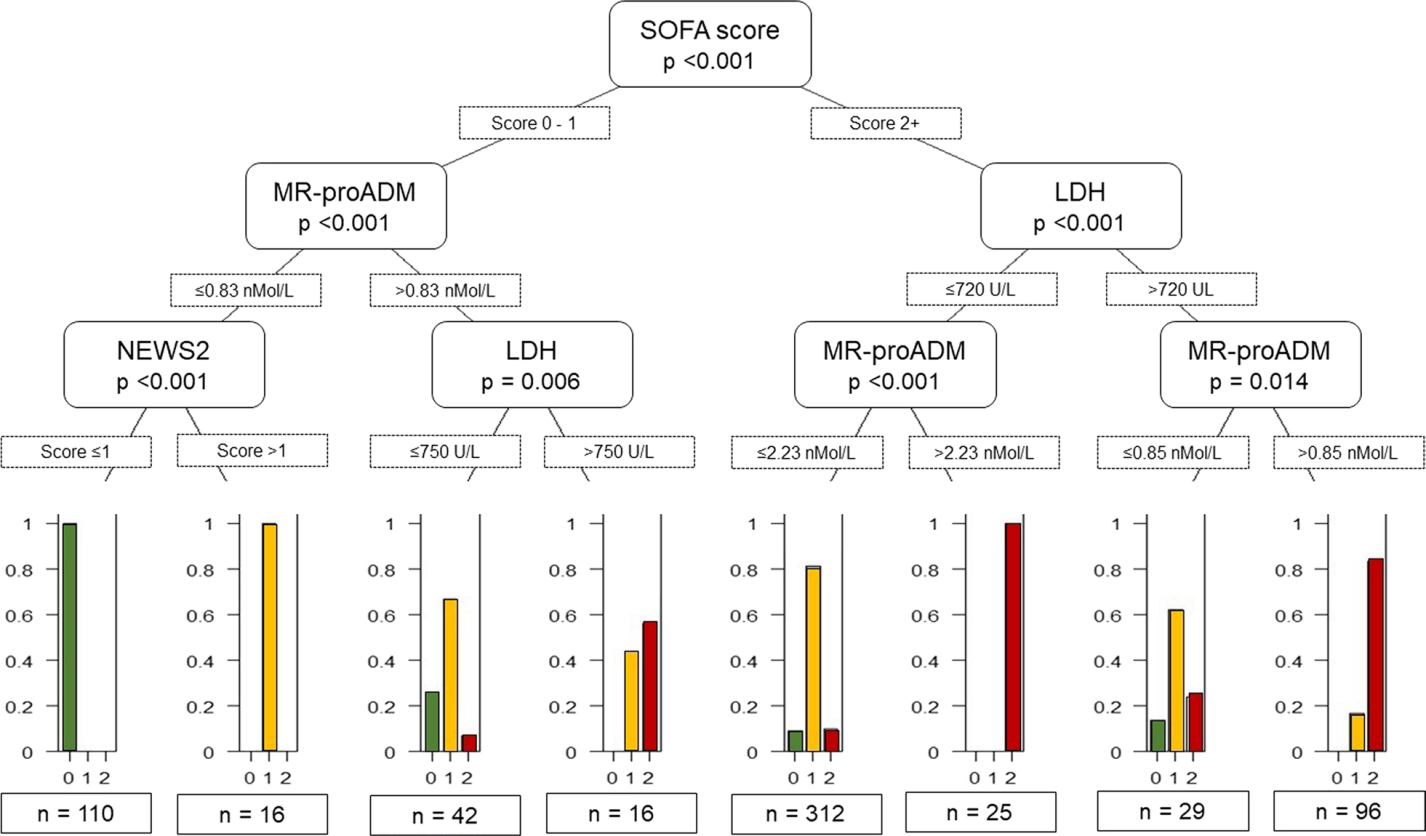
Conditional decision tree developed to explain the predictive performance of the multiclass random forest classifier

The decision tree reported in Fig. 5 allows an interpretation of the most important interactions captured by the random forest classifier. A SOFA score ≥ 2 represents the predominant risk factor in determining the need for hospitalisation, with the predictive performance enhanced by MR-proADM and LDH.

In patients with a SOFA score < 2, if MR-proADM is ≤ 0.83 nmol/L and the NEWS2 score ≤ 1 the probability of being discharged safely is maximum. In patients with a SOFA score < 2, if MR-proADM is > 0.83 nmol/L LDH has significance as a predictor for a poor clinical outcome. Conversely, in patients with a SOFA score ≥ 2 at presentation to ED, if LDH is ≤ 720 U/L but MR-proADM > 2.23 nmol/L the probability of being hospitalised with a negative outcome of death is high. The greatest probability of dying is in those patients with a SOFA score ≥ 2, LDH > 720 U/L and MR-proADM > 0.85 nmol/L.

**Fig. 6:**
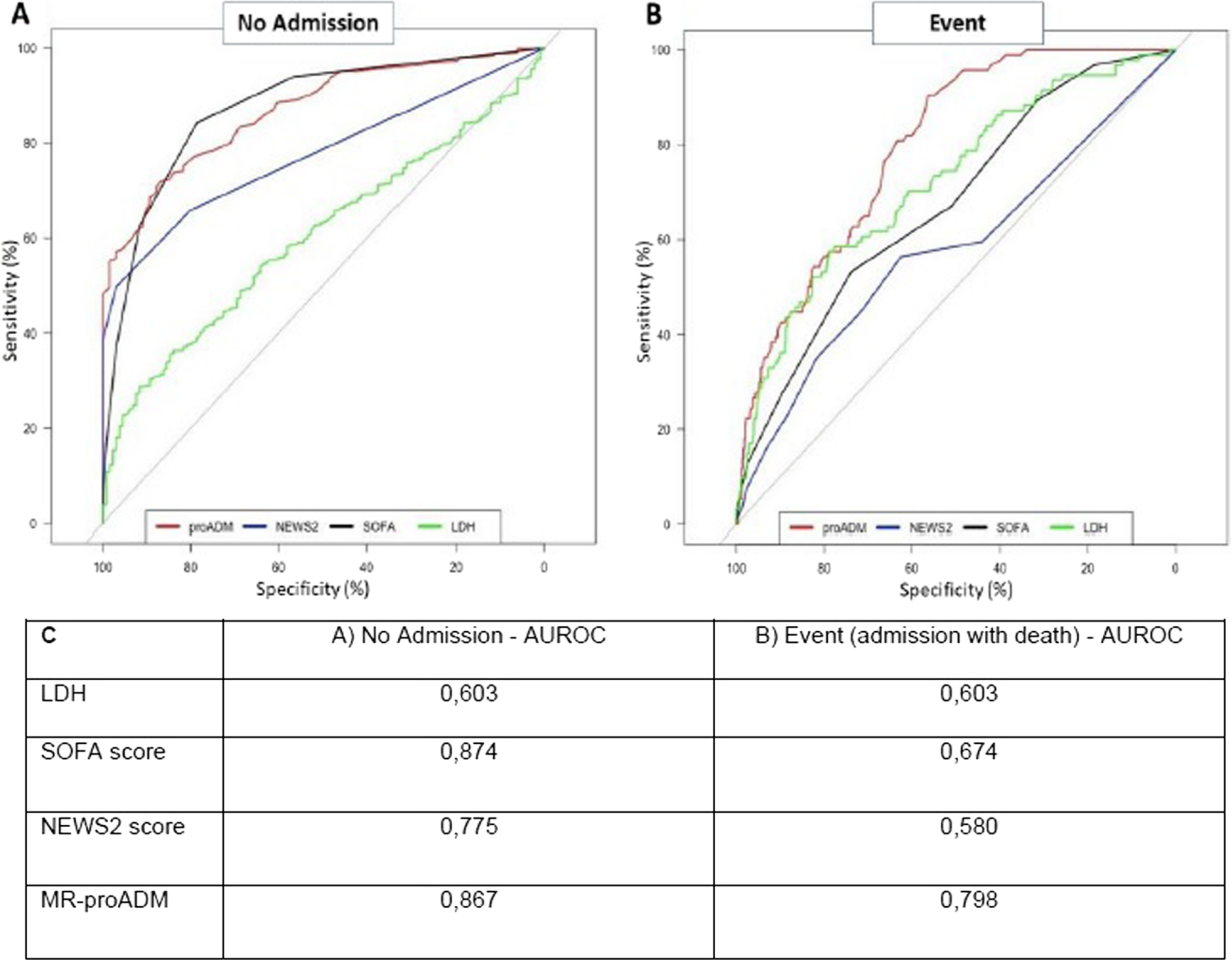
**A** ROC curve for admission avoidance in the subgroup where clinical scores were additionally considered. **B** ROC curve for mortality in the subgroup where clinical scores were additionally considered

The threshold values observed in the surrogate conditional decision tree are partially in agreement with a ROC analysis performed, shown below (Fig. 6), with classic statistical analysis on the biomarkers and on clinical scores:

i. When considering LDH, for non-admitted patients the AUC was 0.603 and the best threshold was 704; for admitted patients who died the AUC was 0.603 and the best threshold was 718.5.
ii. Concerning SOFA scores, the AUC in non-admitted patients was 0.874 and the best threshold was 2, whereas for admitted patients with mortality the AUC was 0.674 and the best threshold was 4.
iii. With regard to NEWS2 score, for non-admitted patients the AUC was 0.775 and the best threshold was 1.5 and for patients admitted who died the AUC was 0.58 and the best threshold was 2.
iv. Regarding MR-proADM, the AUC in non-admitted patients was 0.867 and the best threshold was 0.775, whereas for admitted patients with mortality the AUC was 0.798 and the best threshold was 0.855.

## Discussion

Whilst previous studies have examined the utility of MR-proADM in SARS-CoV-2 patients in determining clinical outcomes these have been small in size, single centre, used different inclusion and exclusion criteria, are often disparate in the clinical outcomes measured and the multivariable regression models used are likely to overfit the predictor effects if standard maximum likelihood estimation (ie. unpenalised estimation) is used [12–14, 16–19, 25, 26]. Several studies have also examined biomarkers and clinical parameters in an attempt to develop algorithms for identifying patients at risk of Intensive Care Unit admission [27–30], however there is a lack of validated clinical scores, algorithms or biomarkers for helping to determine patients appropriate for outpatient management. In this multi-centre retrospective analysis, across 10 sites in Europe, MR-proADM measurement at presentation in combination with other biomarkers or clinical scoring systems could accurately delineate between those in need of admission and those that weren’t as well as determining those at increased risk of all-cause 28-day mortality.

The proposed multiclass random forest classifier models have good statistical performance mainly to identify patients suitable for safe discharge. In fact, for patients that did not require admission the resultant random forest algorithm had a sensitivity of 89.6 ± 2.08%, a specificity of 84.44 ± 2.21% and AUC of 91.14 ± 0.35%, which improved when clinical scores such as SOFA score were added (sensitivity of 93.39 ± 1.53%, specificity of 91.36 ± 1.45% and AUC of 95.9 ± 0.28%).

For patients at high risk of mortality the random forest model was less accurate but still maintains good performance with a sensitivity of 76.02 ± 2.72%, a specificity of 76.8 ± 3.12% and AUC of 81.11 ± 0.37% but in this case, when clinical scores were added it improved the sensitivity but not the specificity and AUC (sensitivity of 85.5 ± 2.86%, specificity of 70.45 ± 3.79% and AUC of 79.37 ± 0.68%).

**Fig. 7:**
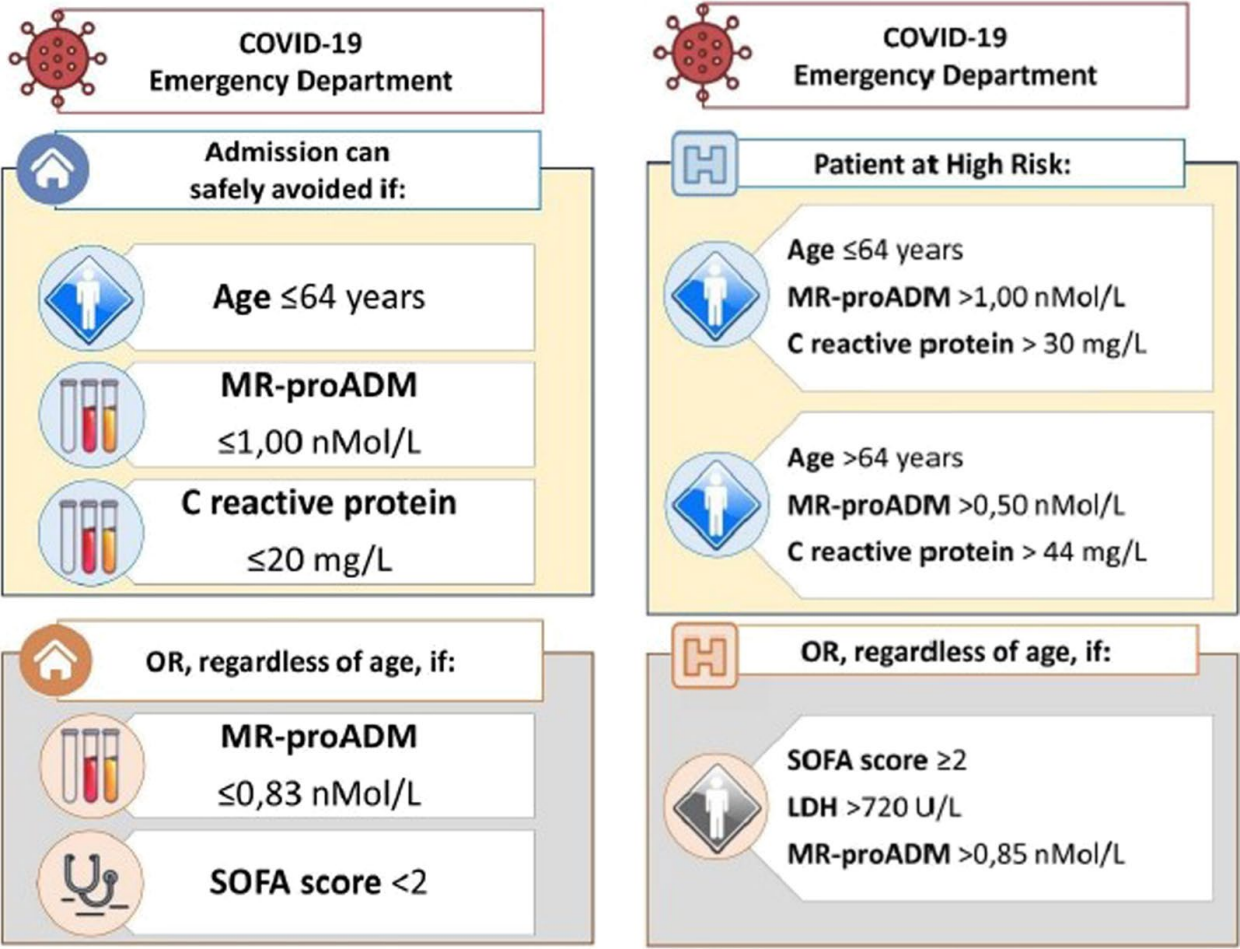
Proposed workflows for managing COVID-19 patients based on results of conditional decision trees. Values presented are rounded for ease of future clinical implementation. Workflows presented are for safe admission avoidance (actual values were: CRP ≤ 20.2 mg/L, MR-proADM ≤ 1.02 nmol/L) and for identifying those at increased risk of mortality (actual values were: CRP > 29.26 mg/L, MR-proADM > 1.02 nmol/L)

On the basis of the results from the conditional decision trees criteria allowing for safe admission avoidance in SARS-CoV-2 patients can be devised, as shown in Fig. 7, where biomarker values are rounded for ease of clinical implementation. Admission may be avoided in patients aged ≤ 64, with an MR-proADM value of ≤ 1.00 nmol/L and a CRP of ≤ 20 mg/L or in patients with an MR-proADM ≤ 0.83 nmol/L and a SOFA score < 2. Figure 7, also provides criteria for patients with an increased mortality risk. In those aged ≤ 64 if their MR-proADM is > 1.00 nmol/L and CRP is > 30 mg/L they should be deemed high risk, as should those aged > 64 if their CRP is 44 mg/L and MR-proADM is > 0.50 nmol/L. Finally, patients with a SOFA score ≥ 2, with an LDH of > 720 U/L and an MR-proADM > 0.85 nmol/L are also at increased risk of mortality.

These threshold values observed in the surrogate conditional decision trees and from the thresholds derived from the ROC analyses (0.775 nmol/L when incorporating SOFA and NEWS2 scores or 0.771 nmol/L when these were not taken in to consideration) for determining patients suitable for discharge from ED are broadly consistent with previous studies examining patients with bacterial infections. Albrich et al. found that outcomes were substantially improved for patients with a MR-proADM of ≤ 0.75 nmol/L and CURB-65 of 0–1 [31]. MR-proADM levels of < 0.80 nmol/L in patients presenting with urinary tract infections were shown to be effective at identifying patients who could be safely managed as outpatients.[32] A derived cut-off of < 0.87 in patients presenting to emergency departments could identify patients for outpatient management without an increase in 28 day mortality or readmission [7].

SARS-CoV-2 causes a viral sepsis[33–35] and as such the results presented here are concordant with the new definition of sepsis[36] that incorporates a SOFA score of ≥ 2; the optimal threshold SOFA score for delineating between non-admission and admission was 2, (see Figs. 5 and 6A).

The finding that MR-proADM has the greatest importance in the random forest model presented here could be explained, in part, by its kinetic profile, which is rapidly produced relative to CRP and PCT [37], consistent with previous studies identifying MR-proADM as more accurate than CRP and PCT in identifying disease severity and treatment response [6]. As endothelial dysfunction secondary to infection progresses towards multiple organ dysfunction and subsequent failure [38], MR-proADM may provide a convenient measure for the early identification of potential disease progression [39]. This is particularly pertinent during SARS-CoV-2 infection due to the endotheliitis induced, resulting in complications such as thromboembolism, vascular disease and acute respiratory distress syndrome.

This is the largest study examining MR-proADM in SARS-CoV-2 patients and, as such, the interpretation of results here is not restricted by the same limitations placed on studies prior to this, such as previous studies being at risk of over-fitting their models. However, there are several limitations, this model does not account for treatments validated in the management of COVID-19 such as immunomodulators or interleukin-6 inhibitors, due to limitations in the methods of data collection employed at some sites. It also remains to be seen whether the application of novel assays into clinical diagnostic and management pathways will deliver the potential expected benefits since clinician confidence has to be developed over time. Before this novel assay can be implemented into routine clinical practice the evaluation of associated health economic data would also be advisable.

## Conclusion

This is the first large multicentre study examining the prognostic utility of MR-proADM in a population with viral infection, in this case SARS-CoV-2, in predicting need for admission from the Emergency Department and in predicting mortality. The measurement of a standardised set of biomarkers and clinical parameters, that includes MR-proADM, CRP, LDH upon presentation, in patients infected with SARS-CoV-2, could help identify those that are suitable for discharge from ED, when interpreted in the context with the cut-off values presented here. Conversely, these measurements may also be used to identify patients with an increased mortality risk. As such, the incorporation of MR-proADM into a management protocol may improve outcomes and patient care pathways.

## Data Availability

Data is available upon reasonable request to the corresponding authors.

## Abbreviations

ADM: Adrenomedullin
AUC: Area under the curve
CRP: C-Reactive Protein
EDTA: Ethylenediaminetetraacetic acid
ED: Emergency Department
LDH: Lactate Dehydrogenase
MR-proADM: Mid-Regional proAdrenomedullin
NEWS2: National early warning score 2
PCT: Procalcitonin
RT-qPCR: Real‐time reverse-transcription PCR
ROC: Receiver operating characteristic
SARS-CoV-2: Severe Acute Respiratory Syndrome Coronavirus-2
SOFA: Sequential organ failure assessment
WCC: White blood cell count
